# Supplementary material for: Rest Induces a Distinct Transcriptional Program in the Nervous System of the Exercised L. stagnalis
Source: Int J Mol Sci. 2025 Jul 20;26(14):6970. doi: 10.3390/ijms26146970 (PMC12294990; doi:10.3390/ijms26146970)
Supplement: Supplementary file 1 [file ijms-26-06970-s001.zip › ijms-3715705-supplementary figures.pdf]

Supplementary materials description

Supplementary Figures S1–S4

Containы supplementary figures.

Transcripts_expr.csv

Contains transcripts related to Figure 2 and Supplemental Figure S1 with corresponding Log_2(FC), FDR, p-values, protein identities and annotations.

Mouse-Snail.csv

Genes regulated by rest or exercise in snails and after voluntary exercise in mice.

Supplementary figures

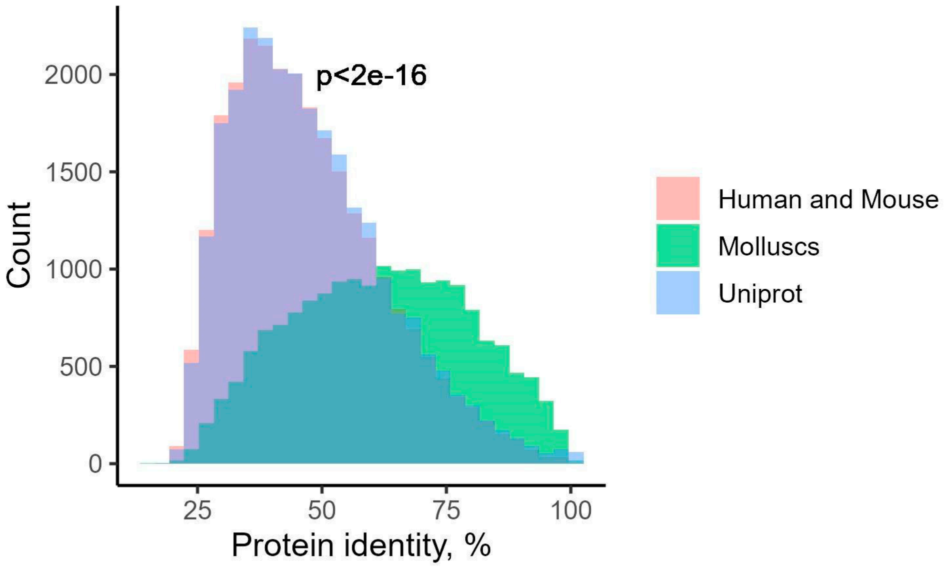

Supplemental Figure S1. Using Uniprot or human and mouse reference sequences allowed identification of more proteins although with lower average percent identity in comparison to the reference combined from sequences of 6 molluscs. Distribution of identity percent between blast results for Uniprot database, Human and mouse reference sequences or reference sequences from the 6 molluscs (Lottia gigantea (taxid: 225164), Elysia chlorotica (taxid: 188477), Mizuhopecten yessoensis (6573), Crassostrea gigas (taxid: 29159), Mytilus coruscus (taxid: 42192), Pomacea canaliculata (taxid: 400727)). p - represents Kolmogorov-Smirnof test.

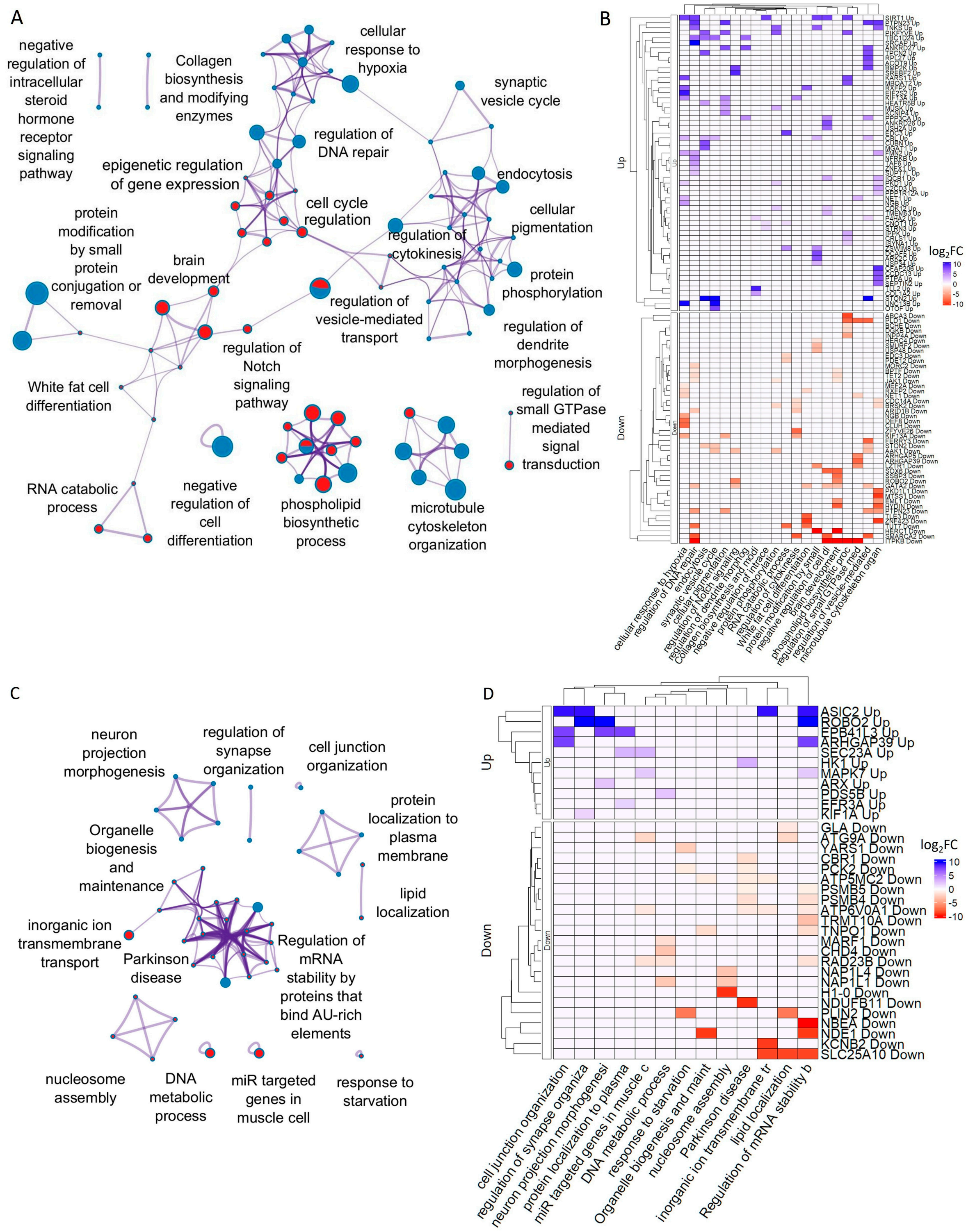

Supplementary Figure S2. Annotation of genes regulated by exercise or rest with FDR<0.05. A, C. Clustering of annotations overrepresented in the lists of genes regulated by exercise (A) or rest (C). B, D. Clustering of genes regulated by exercise (B) or rest (D) and corresponding annotations. Colors represent log2(Fold Change) relative to control animals.

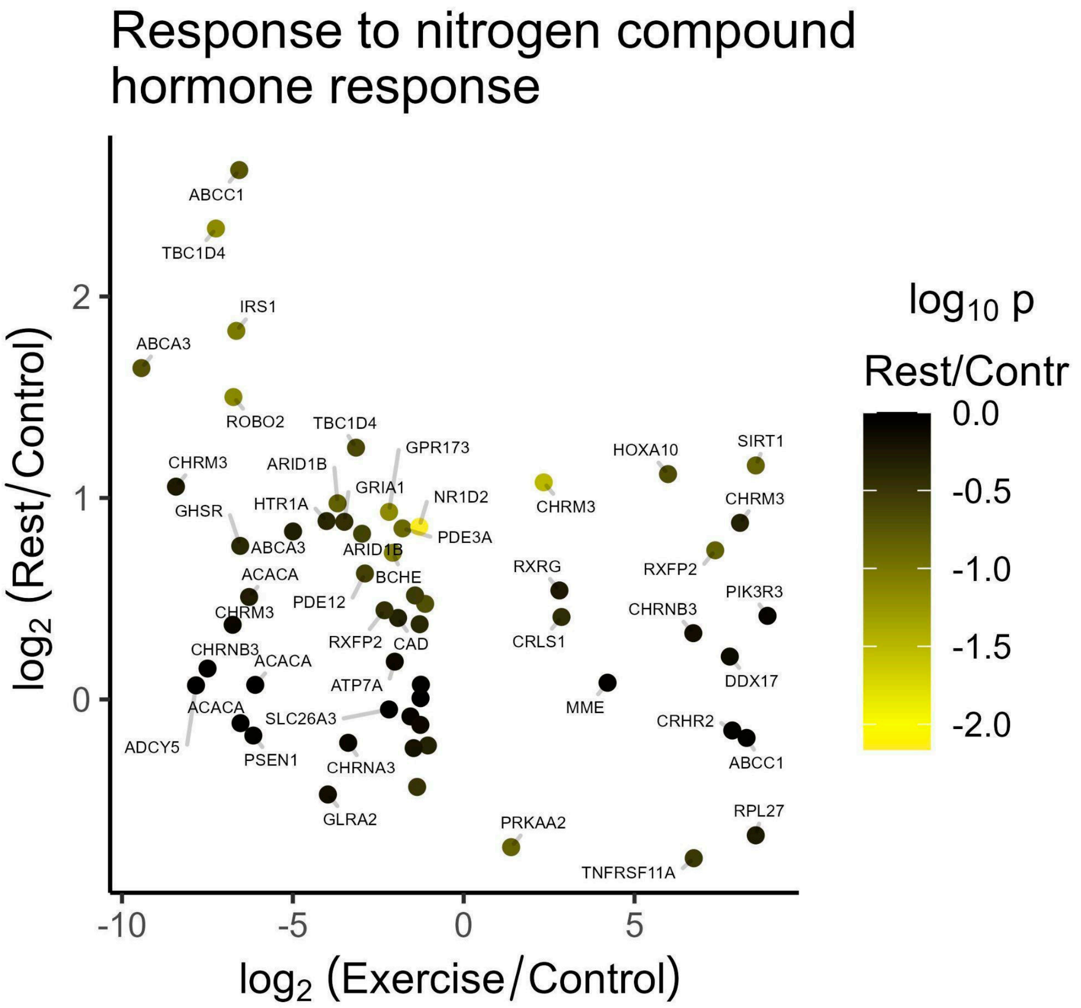

Supplemental Figure S3. Changes of transcripts from the response to nitrogen compound cluster that are mostly repressed upon exercise revealed a set of genes that change the most. Colors represent $\log_{10}$(p-values) for changes of gene expression in the rested relative to the control animals. The majority of these genes do not change significantly in rested relative to the control animals.

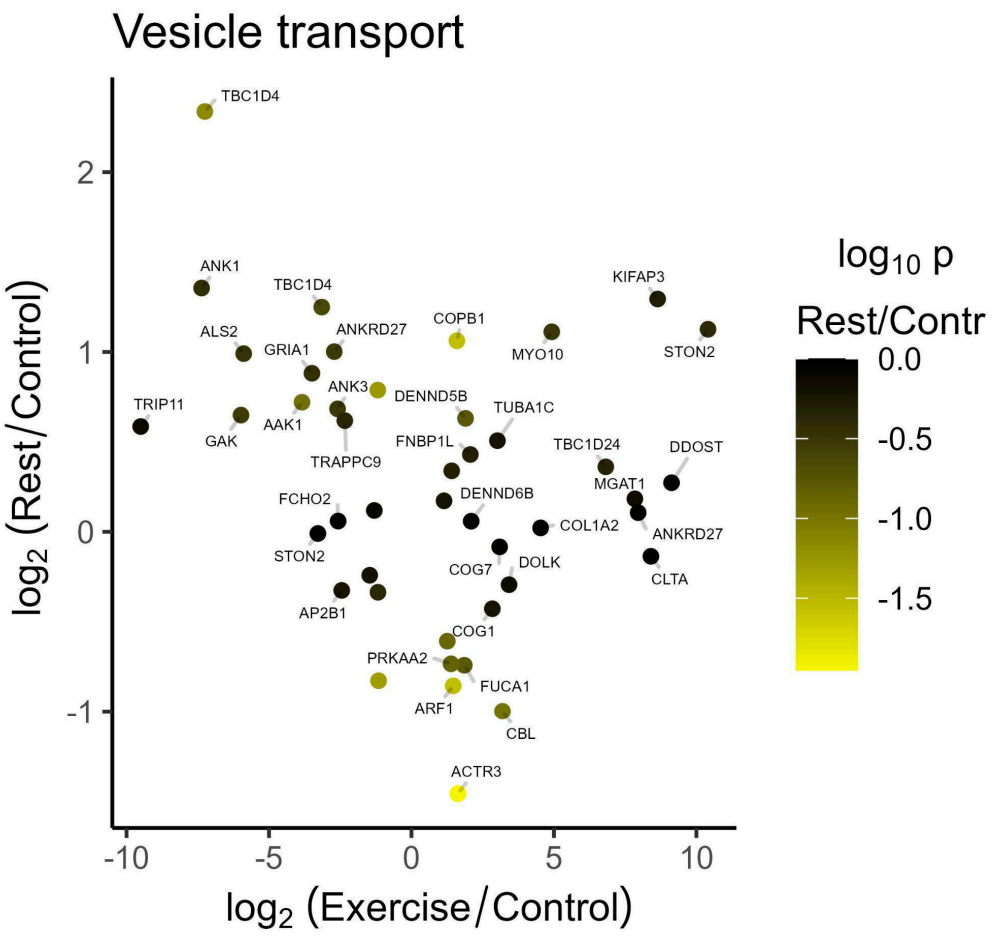

Supplemental Figure S4. Changes of transcripts from the vesicle transport clusters that are mostly induced upon exercise revealed a set of genes that change the most. Colors represent $\log_{10}$(p-values) for changes of gene expression in the rested relative to the control animals. The majority of these genes do not change significantly in rested relative to the control animals.
